# Supplementary material for: Effectiveness of unguided internet-based computer self-help platforms for eating disorders (with or without an associated app): A systematic review
Source: PLOS Digit Health. 2025 Apr 23;4(4):e0000684. doi: 10.1371/journal.pdig.0000684 (PMC12017517; doi:10.1371/journal.pdig.0000684)
Supplement: Attachment — Submitted filename: Response to Reviewers.docx [file pdig.0000684.s003.docx]

**Dear Editor and Reviewers,**

We appreciate your feedback and expertise on this manuscript.

We revised according to your feedback as described below:

**Editor:**

1. We ask that a manuscript source file is provided at Revision. Please upload your manuscript file as a .doc, .docx, .rtf or .tex.

- *We uploaded the doc file for the revised version.*

2. Please provide separate figure files in .tif or .eps format.

For more information about figure files please see our guidelines:
<https://journals.plos.org/digitalhealth/s/figures> 
<https://journals.plos.org/digitalhealth/s/figures#loc-file-requirements>

- *We provided the figure files in .tif format*

3. In the online submission form, you indicated that "The results of the datasets used in this systematic review are available from the corresponding author upon reasonable request.".

All PLOS journals now require all data underlying the findings described in their manuscript to be freely available to other researchers, either 
a. In a public repository, 
b. Within the manuscript itself, or 
c. Uploaded as supplementary information.

This policy applies to all data except where public deposition would breach compliance with the protocol approved by your research ethics board. If your data cannot be made publicly available for ethical or legal reasons (e.g., public availability would compromise patient privacy), please explain your reasons by return email and your exemption request will be escalated to the editor for approval. Your exemption request will be handled independently and will not hold up the peer review process, but will need to be resolved should your manuscript be accepted for publication. One of the Editorial team will then be in touch if there are any issues.

- *The results of the datasets are provided in the supplement 1. We added the information on line 212 “Supplement 1 shows the data search results.”.*

4. We have amended your Competing Interest statement to comply with journal style. We kindly ask that you double check the statement and let us know if anything is incorrect.

- *We confirm the statement is correct.*

5. As required by our policy on Data Availability, please ensure your manuscript or supplementary information includes the following:

A numbered table of all studies identified in the literature search, including those that were excluded from the analyses.

For every excluded study, the table should list the reason(s) for exclusion.

If any of the included studies are unpublished, include a link (URL) to the primary source or detailed information about how the content can be accessed.

A table of all data extracted from the primary research sources for the systematic review and/or meta-analysis. The table must include the following information for each study:

Name of data extractors and date of data extraction

Confirmation that the study was eligible to be included in the review.

All data extracted from each study for the reported systematic review and/or meta-analysis that would be needed to replicate your analyses.

If data or supporting information were obtained from another source (e.g. correspondence with the author of the original research article), please provide the source of data and dates on which the data/information were obtained by your research group.

If applicable for your analysis, a table showing the completed risk of bias and quality/certainty assessments for each study or outcome.  Please ensure this is provided for each domain or parameter assessed. For example, if you used the Cochrane risk-of-bias tool for randomized trials, provide answers to each of the signalling questions for each study. If you used GRADE to assess certainty of evidence, provide judgements about each of the quality of evidence factor. This should be provided for each outcome.

An explanation of how missing data were handled.

This information can be included in the main text, supplementary information, or relevant data repository. Please note that providing these underlying data is a requirement for publication in this journal, and if these data are not provided your manuscript might be rejected.

- *We have included the number of analysed studies (Fig 1), data search terms and results (Fig 1 and Supplement 1), reasons of excluding studies (Fig 1), table of extracted data (Table 2), name of data extractors and date (Methods section), eligibility criteria (Methods section), all extracted data (Supplement 1), date of corresponding with original research author (Drop-out rates subsection), risk of bias and GRADE information (Fig 2, Fig 3, Table 3).*

**Additional Editor Comments (if provided):**

1. It is a bit unclear what is meant by “computer-based platforms.” Are these mobile apps? If so, when the authors say “Computer-based self-help platforms ± associated apps,” it would just mean “app platforms + associated apps,” which is redundant. If these are not solely mobile apps, what other types of computers are being discussed? Desktop computers, tablet computers, or something else?

   Regarding the first comment above, it is not merely about the use of terminology, but it also impacts the screening and selection of studies to be included in the review. Specifically, if the term "computer-based platforms" was not clearly defined from the beginning, it would have affected the screening and selection process conducted by the researchers.

- *Prior to publishing the protocol, JMIR reviewers requested the use of computer-based self platforms ± apps throughout the manuscript; hence our term used. Considering PLoS reviewers feedback, we are happy to amend the terminology to internet-based computer self-help platforms.*
- *While smartphone apps are a growing area of research in eating disorder interventions (e.g., Tregarthen et al., 2019; Juarascio et al., 2015), this review specifically focuses on computer-based platforms to provide a distinct analysis of their unique features and impact. Definition clarification (also added to introduction):* *We define internet-based computer self-help platforms as interventions designed to be accessed via desktop or laptop computers which need access to the internet. These platforms may, at times, be accompanied by smartphone apps, which serve as supplementary tools but are not standalone interventions.*

*Several studies included in this review examined platforms combining computer-based interventions with supplementary smartphone apps. These studies were included only if the primary intervention was computer-based, and the apps served as adjunct tools rather than standalone solutions.*

- *We also added the following points to inclusion/exclusion table:*

*Inclusion:*

- *Interventions delivered primarily via internet-based computer self-help platforms or websites, regardless of whether they were accompanied by supplementary smartphone apps.*
- *Interventions targeting eating disorder symptoms or behaviors, delivered through structured, self-guided programs designed for personal computers*

*Exclusion:*

- *Studies focusing exclusively on standalone mobile smartphone applications as the primary intervention.*
- *Studies that did not specify the platform or delivery method of the intervention.*
- *Discussion changes: rationale for not including mobile apps.*

*The decision to exclude studies focusing solely on standalone mobile smartphone applications was made to provide a distinct analysis of computer-based self-help platforms and their unique contribution to eating disorder interventions. However, it is acknowledged that smartphone apps represent a rapidly growing area of research in this field. Mobile app self-help platforms provide unique advantages primarily surrounding commodity and accessibility which may have serious implications for the future of computer-based self-help platforms which are not accompanied by a mobile app version. Future research should compare mobile and computer-based self-help platforms for EDs to better understand the respective strengths, limitations, and potential synergies for ED treatment.*

2. In Table 2, for each study, please provide information about what computer technology was involved. I believe this information can be presented in the column titled “Intervention and comparison group(s).” However, if it is not appropriate there, the authors can include a new column for it. For example, in Luo et al., 2021, the intervention is described as “Psychoeducation,” but no information is provided about the technology used to deliver the psychoeducation. Please include such information for all studies in Table 2.

- *We added the information in Table 4 in the platform & aim column. This table will give the readers a clearer view of the discussed modalities.*

**Reviewer #1**

1. Did you exclude studies on mobile smartphone apps? If so, there needs to be rationale for this. Additionally, computer-based self-help platforms +/- associated apps are mentioned, but does this mean smartphone apps? Please clarify in the manuscript. This implies that some studies include a computer-based platform as well as an app, but there isn’t stratification or discussion around the difference between computer-based platforms alone vs with apps. Smartphone apps do need to be addressed here, there are many studies on smartphone apps for eating disorders (Tregarthen J, Kim JP, Sadeh-Sharvit S, Neri E, Welch H, Lock J. Comparing a tailored self-help mobile app with a standard self-monitoring app for the treatment of eating disorder symptoms: Randomized controlled trial. JMIR Mental Health. 2019 Nov 21;6(11):e14972, Juarascio AS, Manasse SM, Goldstein SP, Forman EM, Butryn ML. Review of smartphone applications for the treatment of eating disorders. European Eating Disorders Review. 2015 Jan;23(1):1-1.)

- *Prior to publishing the protocol, JMIR reviewers requested the use of computer-based self platforms ± apps throughout the manuscript; hence our term used. Considering PLoS reviewers feedback, we are happy to amend the terminology to internet-based computer self-help platforms.*
- *While smartphone apps are a growing area of research in eating disorder interventions (e.g., Tregarthen et al., 2019; Juarascio et al., 2015), this review specifically focuses on computer-based platforms to provide a distinct analysis of their unique features and impact. Definition clarification (also added to introduction): We define internet-based computer self-help platforms as interventions designed to be accessed via desktop or laptop computers which need access to the internet. These platforms may, at times, be accompanied by smartphone apps, which serve as supplementary tools but are not standalone interventions.*

*Several studies included in this review examined platforms combining computer-based interventions with supplementary smartphone apps. These studies were included only if the primary intervention was computer-based, and the apps served as adjunct tools rather than standalone solutions.*

- *We also added the following points to inclusion/exclusion table:*

*Inclusion:*

a. *Interventions delivered primarily via internet-based computer self-help platforms or websites, regardless of whether they were accompanied by supplementary smartphone apps.*
b. *Interventions targeting eating disorder symptoms or behaviors, delivered through structured, self-guided programs designed for personal computers*

*Exclusion:*

c. *Studies focusing exclusively on standalone mobile smartphone applications as the primary intervention.*
d. *Studies that did not specify the platform or delivery method of the intervention.*
- *Discussion changes: rationale for not including mobile apps.*

*The decision to exclude studies focusing solely on standalone mobile smartphone applications was made to provide a distinct analysis of computer-based self-help platforms and their unique contribution to eating disorder interventions. However, it is acknowledged that smartphone apps represent a rapidly growing area of research in this field. Mobile app self-help platforms provide unique advantages primarily surrounding commodity and accessibility which may have serious implications for the future of computer-based self-help platforms which are not accompanied by a mobile app version. Future research should compare mobile and computer-based self-help platforms for EDs to better understand the respective strengths, limitations, and potential synergies for ED treatment.*

2. The sentences in lines 124-126 belong in the methods section

- *We have removed the sentences in lines 124-126, and the detailed information has been already included in the methods section.*

3. Is there a reference for the protocol that was “published on JIMR journal” (line 132)?

- *We have added the protocol reference: Gentile A, Kristian YY, Cini E. Effectiveness of Computer-Based Psychoeducational Self-Help Platforms for Eating Disorders (With or Without an Associated App): Protocol for a Systematic Review. JMIR Res Protoc. 2024;13:e60165.*

4. Searching databases from inception onwards was mentioned in the abstract, but not in the search strategy section of the methods. Please add this to the methods section, and consider also putting in brackets behind each database the year of inception.

- *We added the information in the search strategy: Four databases, Ovid MEDLINE I (inception: 1946), Embase (inception: 1974), Global Health (inception: 1910), and APA PsychInfo (inception: 1806), were used to identify relevant literature from inception to 31 May 2024.*

5. Consider also including a full search from one database as a supplementary file so readers can see exactly what terms and subject headings you used.

- *We provided the full search from the databases in Supplement 1.*

6. Consider using PICOS (population, intervention, comparator/control, outcome, study design) to organize rows on your eligibility criteria table, it was slightly difficult to follow.

- *We have revised the eligibility criteria subsection with the PICOS format*

7. You can remove this sentence (line 147-148): “The systematic review excluded literature that did not meet the inclusion criteria.” It is implied that studies need to meet inclusion criteria.

- *We have removed the sentence.*

8. Please change the wording the reason for excluding reports so that it’s consistent with your exclusion criteria. I’m not sure what “Not appropriate reporting style” means. Does this refer to publication type or study design? Or something else?

- *We changed the wording in the PRISMA flow chart to “different study design”, to make it more consistent with the exclusion criteria.*

**Reviewer #2:**

1. The title "Clinical Effectiveness of Computer-Based Psychoeducational Self-Help Platforms for Eating Disorders (with or without an associated App): A Systematic Review" is misleading. The population included are not all clinical in nature. As noted, the studies span primary, secondary, and tertiary interventions thereby including sub-clinical populations. The authors appear interested not only in whether an individual meets criteria for an ED, but also premorbid indicators.

- *We removed the term ‘clinical’ in the title to avoid misinterpretation. We also changed the term ‘Computer-Based Psychoeducational Self-Help Platforms’ to minimise confusion. In accordance with feedback from editors and reviewers, we changed our title to: “Effectiveness of unguided internet-based computer self-help platforms for eating disorders (with or without an associated app): A systematic review”*

2. Regarding the title and the paper overall, the use of the term "computer-based' is inaccurate in my view. Computer based could mean with or without the use of the internet. It is my understand that all of these interventions are internet, online, or web-based. Therefore those terms are more accurate. Computer-based could mean an external program downloaded to a computer and used offline, or in earlier tech the use of a CD-Rom program. It is important in describing these programs to use the most accurate terminology to avoid confusion.

- *We only include unguided web-based and internet-based computer programs. We described the definition of this term and added the necessary information in the introduction, methods, and discussion to avoid confusion.*
- *While smartphone apps are a growing area of research in eating disorder interventions (e.g., Tregarthen et al., 2019; Juarascio et al., 2015), this review specifically focuses on computer-based platforms to provide a distinct analysis of their unique features and impact. Definition clarification (also added to introduction): We define internet-based computer self-help platforms as interventions designed to be accessed via desktop or laptop computers which need access to the internet. These platforms may, at times, be accompanied by smartphone apps, which serve as supplementary tools but are not standalone interventions.*

*Several studies included in this review examined platforms combining computer-based interventions with supplementary smartphone apps. These studies were included only if the primary intervention was computer-based, and the apps served as adjunct tools rather than standalone solutions.*

- *We also added the following points to inclusion/exclusion table:*

*Inclusion:*

- *Interventions delivered primarily via internet-based computer self-help platforms or websites, regardless of whether they were accompanied by supplementary smartphone apps.*
- *Interventions targeting eating disorder symptoms or behaviors, delivered through structured, self-guided programs designed for personal computers*

*Exclusion:*

- *Studies focusing exclusively on standalone mobile smartphone applications as the primary intervention.*
- *Studies that did not specify the platform or delivery method of the intervention.*
- *Discussion changes: rationale for not including mobile apps.*

*The decision to exclude studies focusing solely on standalone mobile smartphone applications was made to provide a distinct analysis of computer-based self-help platforms and their unique contribution to eating disorder interventions. However, it is acknowledged that smartphone apps represent a rapidly growing area of research in this field. Mobile app self-help platforms provide unique advantages primarily surrounding commodity and accessibility which may have serious implications for the future of computer-based self-help platforms which are not accompanied by a mobile app version. Future research should compare mobile and computer-based self-help platforms for EDs to better understand the respective strengths, limitations, and potential synergies for ED treatment.*

3. Again referring to the title and the paper throughout, I found it confusing that the authors continually used the plus-minus sign to with "associated apps". The plus-minus symbol was distracting to me throughout the paper. I recommend again using a term such as web-based or online or Internet based.

- *Prior to publishing the protocol, JMIR reviewers requested the use of computer-based self platforms ± apps throughout the manuscript; hence our term used. Considering PLoS reviewers feedback, we are happy to amend the terminology to internet-based computer self-help platforms.*
- *To simplify the term and avoid confusion, we defined it as an ‘internet-based computer self-help platform’, which includes web-based and online computer programs and could be accompanied by their associated mobile apps.*

4. I also believe it would be important to describe which of these interventions were browser based only, app based only, or amiable on both? Perhaps there are different drop out rates when a program is also available via an app.

- *We added the information on the intervention in Table 4 in the Platform & Aim column. We found most studies, regardless of the interventions, have high dropout rates, with only two studies with low dropout rate: one was found in the web-based intervention, the other was found in the internet-based computer platform. We did not find significant dropout rate differences between both interventions.*

5. It also seems the authors interchangeably use "self-help programs" along with their use of "computer based programs" to refer to online programs, but this is to be inferred by the reader. Again this creates confusion. A 'self help program' is not by definition computer based or internet based. Please use care and clarity in writing.

- *We differentiated the ‘self-help programs’ and ‘computer-based programs’ and added subsections in our introduction to clarify the terms used in the manuscript.*

6. I found the introduction literature review to be a bit disorganize with key information combined without a clear structure or guiding framework. For example, self-help programming's ability to overcome barriers is included in the same paragraph as the impact of COVID on use of interventions as well as description of the 'waiting well'. I recommend re-working the introduction focusing on flow and organization and improved used of headings and subheadings.

- *We reorganize the introduction and used headings to make it clearer for the readers.*

7. While space may not allow for a thorough description, I think it would be helpful to provide more information and research supporting the key outcomes measured - thin idealisation, body dissatisfaction, quality of life, depression, perseverative thinking, resistance to change. Perhaps earlier in the introduction in describing Eds, the core components and cognitive/affective risk factors.

- *We added short information regarding this in the introduction: “Core components include thin idealization, which refers to the societal and personal endorsement of an unrealistically slim body type as the ideal standard of beauty [3]. This often leads to body dissatisfaction—a pervasive sense of unhappiness with one's physical appearance—that is a strong predictor of ED onset [4]. Additional psychological factors such as depression, perseverative thinking (repetitive, negative thought patterns), and resistance to change also play significant roles in both the aetiology and progression of EDs [5]. These elements not only impair quality of life but also create barriers to recovery, reinforcing the urgency for targeted and accessible interventions [6]. Early identification and addressing these factors through psychoeducation and evidence-based interventions are critical in mitigating the risks associated with EDs.”*

8. Line 124 - 126 information should be in the method section.

- *We have removed the sentence and ensured the information has been included in the methods section.*

9. 1 Line 62-66 is an awkward long sentence. Recommend rewording.

- *We changed the sentence: “EDs have been demonstrated to negatively impact the psychological, cognitive, physical, and social development of individuals. This evidences the need for accessible resources which target early identification through psychoeducation, such as self-help platforms. Self-help platforms support individuals through early intervention and resource signposting.”*

10. The authors are clear in their intention to include those at risk of developing an ED but do not provide what criteria indicate this population of individuals. The reader infers this by reviewing closely the study characteristic table. Preferably the authors will include this information in their method section at the very least, and preferably also in the introduction setting up the rational for including the population of interest.

- *We included the population at risk criteria in the Methods subsections: ‘Eligibility criteria—Population’ and ‘Strategy for data synthesis’.*
- *We integrated the rationale for the population criteria in several subsections in the Introduction: ‘ED treatment’, ‘Computer-based self-help platforms’, and ‘Aim’.*

11. Line 160 is a typo in "were involved participants"

- *We revise the sentence to: “Based on the stepped and THRIVE approach, studies included in the primary prevention were participants from the general population.”*

12. Line 167, please provide a citation for GRADE

- *We have added the GRADE citation: Guyatt, G.H., Oxman, A.D., Vist, G.E., Kunz, R., Falck-Ytter, Y., Alonso-Coello, P., Schünemann, H.J., GRADE Working Group (2008). 'GRADE: an emerging consensus on rating quality of evidence and strength of recommendations', BMJ, 336, pp. 924.*

13. Inconsistent use of COVID vs. COVID-19. Be consistent.

- *We revised COVID to COVID-19.*

14. Line 366 the authors state that consistent with their findings, existing research shows that higher effectiveness, retention and satisfaction with guided programs vs unguided. While this is true with the overall research base it was not the focus of the reviewed paper therefore the presented paper is unable to say that their findings are consistent.

- *We amended the discussion to emphasise our systematic review focus on unguided internet-based computer self-help platforms.*

*“Further, one study demonstrated higher participant satisfaction and more significant ED symptom reduction was found in the guided internet-based computer self-help platform. Features identified in this systematic review, including engaging with peers, receiving guides from practitioners, and getting reminders improved patients’ experiences during treatment. However, our systematic review does not focus on guided vs unguided internet-based computer self-help platforms, therefore we suggest to review other systematic review in this focus area.”*

15. Similar to the organization of the introduction, I found the discussion section to be scattered, unorganized and at times repetitive. I encourage a thorough edit with an eye toward concise and clear writing.

- *We edited the discussion section and included subsections to provide a better discussion flow.*
